# Physicochemical and Rheological Properties of a Dairy Dessert, Enriched with Chickpea Flour

**DOI:** 10.3390/foods7020025

**Published:** 2018-02-18

**Authors:** Victoria Guadalupe Aguilar-Raymundo, Jorge Fernando Vélez-Ruiz

**Affiliations:** 1 Chemical and Food Engineering Department, Universidad de las Américas Puebla, Ex hacienda Santa Catarina Mártir S/N, San Andrés Cholula, Puebla C.P. 72810, Mexico; victoria.aguilarro@udlap.mx; 2 Programa Académico de Ingeniería Agroindustrial, Universidad Politécnica de Pénjamo, Carretera Irapuato-La Piedad Km 44, Predio El Derramadero, Pénjamo, Guanajuato C.P. 36921, Mexico; 3 FN Consultores, S.A. de C. V. Instituto de Innovación y Desarrollo Tecnológico, Boulevard del Niño Poblano 2901. Unidad Territorial Atlixcayotl, Puebla, Puebla C.P. 72197, Mexico

**Keywords:** chickpea flour, dairy dessert, physicochemical and rheological properties

## Abstract

Dairy desserts are complex mixtures and matrices including main components such as milk, sugar, starch, hydrocolloids, colorants and flavors, with a proteinaceous structure; they are widely consumed and present a semisolid consistency. In this work, the physicochemical and rheological properties of a dairy dessert with the addition of chickpea flour (raw and cooked, at four concentrations) were studied to determine the effect of the flour. The results indicated that luminosity (L*: 62.75–83.29), pH (6.35–7.11) and acidity (1.56–3.56) changed with the type of flour. The flow properties of the custards exhibited a non-Newtonian behavior that was well fitted by three flow models. The studied custard systems were stored for twelve days at 4 °C. The physicochemical and flow properties of the custards changed notably as a function of flour addition and storage time. From all samples, only four were analyzed with oscillatory tests, showing their mechanical spectra with elastic behavior. The dessert texture was also measured, founding that those formulated with Blanco Noroeste chickpea flour exhibited the highest values of hardness (0.356–0.391 N) through the twelve days. It can be concluded that those custard systems with the highest content of flour presented a very good response as a potential new dairy product.

## 1. Introduction

Food product development in which dairy products lead the consumer preference on one side and nutritional improvement needs on the other are important research aspects of Food Science. Dairy desserts are products highly requested and consumed in America, Europe and other countries; they are formulated with a diversity of components, such as milk, carbohydrates, colorants and flavors [1,2,3,4]. The European research action COST 921, suggested a model system consisting of a mixture of milk with starch and carrageenan as the gelling agents [5], in order to standardize a base formulation in which a diversity of modifications and studies have been completed. 

Several studies have been focused on analyzing mixtures of polysaccharides and dairy components to improve the viscosity and consistency of semi solid dairy desserts [6]. Since 2005, Vélez-Ruiz et al. [7], due to its importance and effect on dairy desserts, characterized the rheology of custard model systems to know the influence of milk fat level and several hydrocolloids. Similarly, Tárrega et al. [8,9], analyzed the influence of milk on the rheology of cross-linked waxy maize and tapioca starch dispersions in order to know how these starches contributed to both flow and viscoelastic responses. Starch, as well as hydrocolloids, have been used because they contribute to the consistency and other functional properties of the complex matrix in the custard and other dairy products [7]. González-Thomas et al. [1,2] studied the physicochemical, flow and sensorial characteristics of dairy desserts enriched with inulin as a novel and functional component. Alamprese and Mariotti [10] studied the effect of milk replacers (partially skimmed milk, soy and rice drinks) on the pasting, flow and texture properties of puddings. Toker et al. [3] evaluated the effect of guar and xanthan gums, alginate and carrageenan, as well as their interactions, on flow properties of a dairy dessert. In addition, even though many studies were carried out on the first decade of this century, still there are aspects to be researched, when additional variables, such as ingredients and/or processing, are included or modified; Zapata-Noreña et al. [4] completed a study in which a gum as a prebiotic component was included in some custard dessert formulations. Besides the ingredient modifications, some of the aforementioned studies explored nutritional complementary effects.

The nutritional trend for foods enrichment has been and is very important. However, when new components are incorporated into an existing dairy formulation, the effect of such modification on food properties should be researched. Several related works have been carried out in this decade. Zare et al. [11] incorporated lentil flour at 1–3% (*w*/*v*) in yogurt, in order to evaluate its effect and determined both physical and rheological properties during 28 days of storage. They found that a higher concentration of flour caused an increase in syneresis and also the storage modulus was higher for systems with 3% of flour. Cereal-based desserts, like rice and wheat are popular in Asia, occupying an important place not only due to their taste, but also due to their nutritional quality. Jha et al. [12] developed a process to extend the shelf life of a dairy dessert enriched with dalia (cooked and shredded wheat) and determined its physicochemical properties. Qasem et al. [13] carried out a study of high soluble-fiber pudding by incorporating okra (2–8%) in a dessert formulation, trying to improve both the rheological (flow and texture) and nutritional (soluble fiber) properties of desserts and refer good results for the 2% incorporation level. Additionally, the minimum fiber requirement of Food and Drug Administration (FDA) and good sensory acceptability were reached. When new formulations of dispersed systems as food development are designed, the rheological and physicochemical characterization is very important; Costa et al. [14] researched the rheology nature of a fermented rice extract with a complex starch, fitting the flow response to five mathematical models and concluding that the Power law was the best.

Thus, based on the aforementioned studies related to the incorporation of new components in foods in which there are few studies reporting incorporation of legumes in dairy products, the aim of this study was to develop a dairy dessert incorporating chickpea flour of two types, as a non-conventional flour, to improve the functional and nutritional characteristics of custards, determining their physicochemical and rheological characteristics as well.

## 2. Materials and Methods

### 2.1. Composition and Preparation of the Dairy Dessert

The base formulation (COST) for the custard consists of 88.98 mL of whole milk (3% fat), 6.5 g of sucrose, 0.02 g of *κ*-carrageenan and 4.5 g of starch. For the elaboration of our custard systems, this formulation was considered and the starch was replaced by chickpea flour (taking account of its proximal composition), at different concentrations using two types of flour from two varieties. Raw and cooked flours from Blanco Noroeste (BN) and Costa 2004 (C4) chickpea varieties, and semi-skimmed milk (Svelty, Nestle, Jal., Mexico) were used. Table 1 shows the identification codes for all the formulations, as well as the added flour quantity. 

The method described by Seuvré et al. [15] was followed for the custard preparation. First, powders (flour, sucrose and *κ*-carrageenan) were weighted and dispersed slowly in the correspondent milk volume, for 4 min under constant stirring. Subsequently, the mixture was heated up to 90 °C on a heating plate (Barnstead/Thermolyne, Dubuque, IA, USA) at level 5. When each formulation reached this temperature, it was kept for 5 min and then was allowed at cool down until room temperature. After that, the custard systems were transferred into 100 mL flasks, for refrigeration storage at 4 ± 1 °C for further analysis.

Sixteen custard systems were prepared, following a factorial design: 2 × 2 × 4; with 2 varieties of chickpea (BN and C4), 2 types of flour (raw: R and cooked: C) and 4 concentration levels of flour (8.3%, 9.3%, 10.3% and 11.3%). These concentrations for chickpea flour were computed to replace the starch of the original formulation (4.5 g) by considering only a 50% of starch from the chickpea, and assuming that the other part (50%) of the starch, is inter-acting with the other flour components. These substitutions, in the correspondent formulations, covered a range of 4.15 to 6.65 g of starch from the flour. 

### 2.2. Physicochemical Analysis

Soluble solids were determined at room temperature with a digital refractometer (AR 200 Digital Hand-Held, Reichert, Inc., Depew, NY, USA), the results were given as degrees Brix at 20 °C. The pH values were determined by direct immersion, using a pH meter (Model pH10, Conductronic, Puebla, Mexico) at room temperature. The acidity was measured by the method 947.05 of Association of Official Analytical Chemists (AOAC) [16], based on titration with NaOH. Syneresis was quantified with 10 g of sample by centrifugation, as the percentage of supernatant liquid after centrifugation of the gel during 20 min at 2790 g [17], in a centrifuge Clay Adams (Bellport, NY, USA). The color of the custard was determined with a color meter CR-400 Chroma meter (Konica Minolta Sensing, Inc., Osaka, Japan) using the Commission Internationale de l'Eclairage (CIE Lab) scale, in which the instrument was previously calibrated with a white tile (*Y* = 86.6, *x* = 0.3168, *y* = 0.3242) placing approximately 10 g of sample into the sample plate. The reflectance mode was used for determinations of color parameters. Three replicates were completed for each sample and the experiments were carried out at 0, 4, 8 and 12 days of storage.

### 2.3. Rheological Properties

#### 2.3.1. Flow Behavior

All flow determinations were performed with a digital Brookfield viscometer (DV-III, Brookfield Engineering Laboratories, Inc., Middleboro, MA, USA). The viscometer was adjusted to zero and a spindle LV (a type of viscometer with a specific torque) was set at the instrument. The measuring parameters were determined with the next relationships (1)–(3) from the manufacturer [18]:

(1) $$\gamma =\;\frac{2\omega R_{C}^{2}R_{b}^{2}}{R_{b}^{2}\left(R_{C}^{2}-R_{b}^{2}\right)}$$

(2) $$\omega =\;\frac{2\;\pi N}{60}$$

(3) $$\tau =\;\frac{M\;\left(\frac{reading}{100}\right)}{2\pi R_{b}^{2}L}$$

where: *γ* = shear rate (1/s); *ω* = spindle angular velocity (rad/s); *R_c_* = container radius (m); *R_b_* = spindle radius (m); *N* = spindle speed (rpm, rev/min); *τ* = shear stress (Pa); *L* = spindle height (m); *M* = torque for this viscometer = 6.73 × 10^−5^ N·m. 

The experimental flow responses or rheograms were fitted to three mathematical relationships, Power Law (PL), Herschel–Bulkley (HB) and Bingham plastic (BP) models (Equations (4)–(6), respectively):

(4) $$\mathrm{PL}:\tau =\;K{\dot{\gamma }}^{n}$$

(5) $$\mathrm{HB}:\tau =\tau_{0}+\;K{\dot{\gamma }}^{n}$$

(6) $$\mathrm{BP}:\tau =\;\tau_{0}+\eta {\;}_{p}\gamma .$$

where: *τ* = shear stress (Pa); *K* = consistency coefficient (Pa·s^n^); $\dot{\gamma }$ = shear rate (1/s), *n* = flow behavior index (dimensionless); *τ*_0_ = yield stress (Pa); and *ɳ*_p_ = plastic viscosity (Pa·s). 

Two tests of goodness were applied to fit the obtained experimental data, the percentage of the mean error (PEM, Equation (7)) and the square root of the mean error (RMSE, Equation (8)), to verify the fit of each model to the rheological behavior of each dairy dessert:

(7) $$PEM=\;\frac{100}{n_{e}}\overset{n_{e}}{\underset{i=1}{\sum }}\left(\frac{\tau_{exp}-\tau_{pred}}{\tau_{exp}}\right)$$

(8) $$RMSE=\;\frac{1}{n_{e}}\overset{n_{e}}{\underset{i=1}{\sum }}\left({\left(\tau_{exp}-\;\tau_{pred}\right)}^{2}\right){\;}^{1/2}$$

where: *τ_exp_* = experimental shear stress (Pa); *τ_pred_* = predicted shear stress (from the applied model, Pa); and *n_e_* = the number of experimental data (from the flow curve, dimensionless).

#### 2.3.2. Viscoelastic Behavior

Due to instrumental limitations, only a partial viscoelastic response from the studied systems was carried out, selecting four formulations of the dairy dessert as representative of all. Thus, those systems RBN4, RC44, CBN4 and CC44, including the highest starch content, were characterized by the dynamic performance. Rheological evaluations were completed using a stress-controlled rheometer (ARES RFS-III, TA Instruments, New Castle, DE, USA) on its strain mode, with a plate-plate geometry (lower plate diameter of 34 mm, upper plate diameter of 18 mm and a gap of 12 mm) at constant temperature (20 ± 1 °C). In all of the experiments, 25 mL of the sample were used for the test and the samples were covered with paraffin oil to prevent water loss. The frequency sweep was conducted, with oscillation frequencies ranging from 0.1 to 100 rad/s, in which the storage modulus (G’) and loss modulus (G’’) were measured as a function of frequency. Three replicates were completed for each sample and the experiments were carried out at 1, 5 and 9 days of storage.

#### 2.3.3. Texture Analysis

Texture of the dairy desserts was evaluated by a texture profile analysis (TPA), performed in a Shimadzu texture meter (Model EZ-SX, Shimadzu Corporation, Kyoto, Japan) using a cylindrical probe of 50 mm diameter, with 10 mm of compression, 5 s of waiting time and 2 mm/s of crosshead speed. The experiments were carried out at 0, 4, 8 and 12 days after preparation.

### 2.4. Resistant Starch

Resistant starch (RS) was measured by the method proposed by Goñi et al. [19]. The samples were subjected to a first incubation (40 °C, 60 min, pH 1.5) with pepsin (0.1 mL of 10 mg/mL, Sigma P-7012, Sigma Alimentos, San Pedro, Mexico) for protein removal. A second incubation (37 °C, 16 h, pH 6.9) with α-amylase (1 mL of 40 mg/mL, Sigma A-3176, Sigma Alimentos, San Pedro, Mexico) to hydrolyze digestible starch; a residue treatment with 2 M of KOH for solubilization of the resistant starch, followed by a final incubation (60 °C, 45 min, pH 4.75), with amyloglucosidase (80 µL of 140 U/mL, Sigma A-7255, Sigma Alimentos, San Pedro, Mexico) to hydrolyze the solubilized resistant starch. The determination of glucose was obtained by a glucose oxidase-peroxidase assay, and the RS was calculated as glucose (mg) × 0.9 (conversion factor due to starch hydrolysis). Measurement duplicates were made for each sample.

### 2.5. Statistical Analysis

With exception of the RS test that was completed by duplicate, the rest of the determinations were done in triplicate. All data were subjected to analysis of variance and Tukey test to determine significant differences between the dairy dessert systems, with a confidence level of 95%, using the Minitab software v.16^®^ (Minitab Inc., State College, PA, USA).

## 3. Results and Discussion

All prepared custard systems were analyzed at days 0, 4, 8 and 12, measuring their physicochemical and rheological properties, and days 1, 5 and 9 days for dynamic response. From the obtained results for physicochemical and flow characterization, it was observed that most of the data did not exhibit important or significant changes at intermediate days; therefore, the results presented in this section correspond to 0 (fresh samples) and 12 days of storage, while, for viscoelastic and textural response, all data were included.

### 3.1. Effect on Physicochemical Analysis

The results of the physicochemical characterization of the studied systems are presented in Table 2, only for 0 and 12 days of storage; between these two determinations, notable changes were not observed. The systems with raw flour presented higher values for soluble solids (23.93–27.87 Brix) in comparison with those incorporated with cooked flour (20.40–22.03 Brix), both contents may be considered as acceptable for a balanced food product. These soluble solids represent a fifth part in the custard, and the differences can be attributed to the type of flour obtained by two different treatments. Tárrega et al. [8] reported values of 23.5–28.3 Brix for commercial custards, in a formulation containing adipate, crosslinked starch, gelatin, milk, cream and milk power. 

Regarding the pH, systems with raw flour RC4 showed values of 6.86–7.11, higher than samples containing RBN flour (6.68–6.80) at day 0. Higher pH values that does not follow a general trend are related to the increase in the concentration and type of flour. An interaction between the flour components and milk system could be one of the possible reasons, as well as the solubilization of basic amino acids during cooking to which chickpeas was subjected [20]. These results are also comparable to the range reported by Tárrega et al. [8] with pH values of 6.60–6.81, for commercial vanilla custard. Szwajgier and Gustaw [21] reported pH values of 6.25–6.36 for custards added with different malts. In both cases, although there is some similarity, the formulations are different.

On the other hand, acidity as an important determination for dairy products is related to the presence of organic acids, the acidity values are inversely related to the observed variations in pH values. Those systems with cooked flour had a small increase in acid content (2.12–2.82) and showed a significant difference (*p* < 0.05) due to the formulation, compared to the acidity decrease (1.44–1.80) showed by those systems with raw flour. 

Meanwhile, and as expected, with values between 28% and 55%, the percentage of syneresis was higher in systems with cooked flour (≥43%). In this property, the particular response involves lower number of sites available for protein-water bind due to denaturation of proteins by the heat treatment [17], contributing to the higher syneresis of cooked flour custards. In contrast, systems with prepared raw meal had lower values, with a range of 28–47% and an average of 41, with a reverse relationship, in which the augment in raw flour determined a lower syneresis (more clear for the RC4 flour).

Statistical analysis reveals that concentration of flour had not significant effects (*p* > 0.05) on soluble solids, pH, acidity and syneresis between systems; in contrast, the type of flour generated significant differences (*p* < 0.05) on these parameters.

The corresponding results for the same physicochemical parameters for day 12 are also included in the same Table 2. It is noted that the content of soluble solids of custard type dessert was affected by storage time, showing a significant decrease (*p* < 0.05) for custard formulations prepared with raw flour compared to day 0. 

The pH showed a different response, although the formulations with cooked flours showed a general decreasing trend; on the contrary, those samples with raw flour exhibited an increase in six of the eight custards.

With regard to acidity, the studied systems showed the inverse response. Custards containing raw showed a decrease in acidity while the ones with cooked flour showed an increase from day 0 to day 12 (with only one exception). The syneresis changed significantly (*p* < 0.05) with storage; for samples with raw flour five of them exhibited an augment; in contrast, seven of the systems with cooked flour decreased their water loss or syneresis. Although it is known that the presence of *κ*-carrageenan favors the formation of gels, it was not enough to prevent syneresis of the samples with raw flour; this phenomenon depends on additional factors affecting interactions, such as polymer–polymer, water–polymer, degree of heat treatment, type and solids concentration, pH and some salts [13]. 

### 3.2. Effect on Color

Due to the considerable influence of color for consumer acceptance, it is very important to determine it. The measured color parameters for the studied custards at day 0 are concentrated in Table 3. It is noted that the systems formulated with raw flour of both varieties have high values for brightness (L* > 75) ranging from 75.94–82.93, whereas color parameter a* and b* showed trends toward green (negative values) and yellowness (≥17.09), respectively. On the other hand, custard samples with cooked flour showed values of lower luminosity (64.11–76.23), attributed to the flour processing, particularly to the Maillard reaction; b* showed similar values of yellowness (>18.85), but different values for redness. In particular, systems with CBN showed tendencies towards red unlike those containing CC4, oriented towards green. Concerning the color parameter b*, it is interesting to observe a direct relation between flour concentration and a yellowness increase. 

As expected, some color changes are observed with storage time. At day 12, an important decrease was observed in the parameter L* for fourteen of the sixteen systems, with significant effect of the flour type, similarly for the a* parameter. Analysis of variance reveals that all systems showed significant effect (*p* < 0.05) with respect to the type of flour, and the concentration particularly on the b* parameter. 

The storage time did not significantly affect the a* and b* parameters of the studied systems. Finally, the net change of color as a global color parameter showed a notable range of magnitudes in the formulations (0.63 to 10.95) during the storage period, associated with the type of flour (*p* < 0.05). The color changes are due to the oxidation and darkening reactions by the presence of oxygen. Clearly, a tendency of increase in values ∆E is observed with respect to flour concentration, with an exception made for CBN4.

### 3.3. Flow Behavior Response

**Figure 1:**
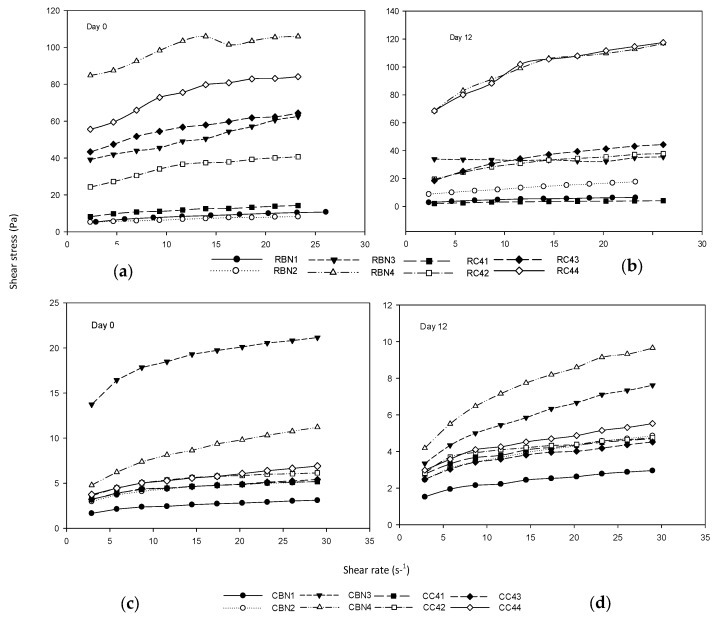
Flows curves of dairy desserts at 0 and 12 days, (**a**) and (**b**) correspond to raw flours (R) and (**c**) and (**d**) correspond to cooked flour (C). BN: Blanco Noroeste; C4: Costa 2004.

Flow curves obtained for the studied systems are shown in Figure 1. The rheograms for all samples showed a non-Newtonian response mainly of plastic and shear thinning nature, exhibiting a yield stress in most of the systems and a characteristic decrease in apparent viscosity with increase in shear rate. These responses are in accordance with other authors who have found a similar behavior for this type of food dispersions.

Those systems made with cooked flour of both varieties, CBN and CC4, exhibited very low stress values (3–21 Pa) compared with those from raw flour systems (5–105 Pa), and a decreasing trend at day 12 in the measured shear stresses for most of the systems. The structural changes in cooked chickpea are combined with the presence of sugar that weaken the initial structure that has been seen in other dispersion systems [22], recording a lower range of 3–10 Pa for shear stress. It was observed, as a different response, that RBN4 and RC44 with higher solids content, implied higher values of shear stress (57–105 Pa), that increased over time to 66–120 Pa, indicating a greater consistency and some degree of structuring. This response is in agreement with other observations for starch–water, starch–milk interactions, in which the structure of the system is affected by an increase in the volume fraction of the dispersed phase consisting of hydrated starch granules. The higher the amount of starch granules, the lower the water absorption and therefore a more consistent and rigid structure is developed [23,24,25]. The other six raw systems (RBN1-RBN3 and RC41-RC43) showed a different and opposite response, with shear stresses from 5–60 at day 0, which decreased to 3–42 Pa with storage (day 12). 

The rheological parameters, such as yield stress (*τ*_0_), flow behavior index (*n*) and consistency coefficient (*K*) obtained from the best fittings for the three applied models, Power Law (LP), Herschel and Bulkley (HB) and Bingham plastic (PB), based on the criterion of the root mean square error (RMSE) for the all systems, are included in Table 4. It is very interesting that the three models were adequate for different samples.

As a very interesting and unusual situation, the flow characterization at both days 0 and 12, show that five systems (RBN1, CBN1, CBN2, CBN3 and CBN4) had a better fit to PL, four of them being cooked and the other with the lowest level of raw flour. Seven systems (RBN4, RC41, RC42, RC43, CC41, CC43 and CC44) showed a better fitting to HB, being four dispersions with raw and three with cooked flour, while the rest of the systems (RBN2, RBN3, RC44 and CC42) were best fitted by BP. Therefore, most of the systems exhibited a yield stress (eleven of sixteen desserts). The systems added with cooked flour showed lower yield stress values than those systems added with raw flour. The differences are due to the type of flour and solid concentration affecting the flow response of the complex mixture of components of this particular dairy product.

The consistency coefficient (*K*), from HB and LP models in freshly prepared systems (day 0) ranged from 0.33 to 11.76 Pa s^n^, and a decrease at day 12 is clearly observed in most of these dessert systems (0.09–2.91 Pa s^n^). On the other side, those values obtained in this study for flow index (*n*) are in agreement with the range 0.35 to 0.60 (PL model) found by Tárrega and Costell [26] for dairy desserts with added starch; Gonzalez-Tomas et al. [1,2] also reported *n*-values (0.20–0.40, PL model) for desserts made with different types of inulin and milk, lower *n*-values indicating “a more” non-Newtonian nature.

### 3.4. Viscoelastic Response

**Figure 2:**
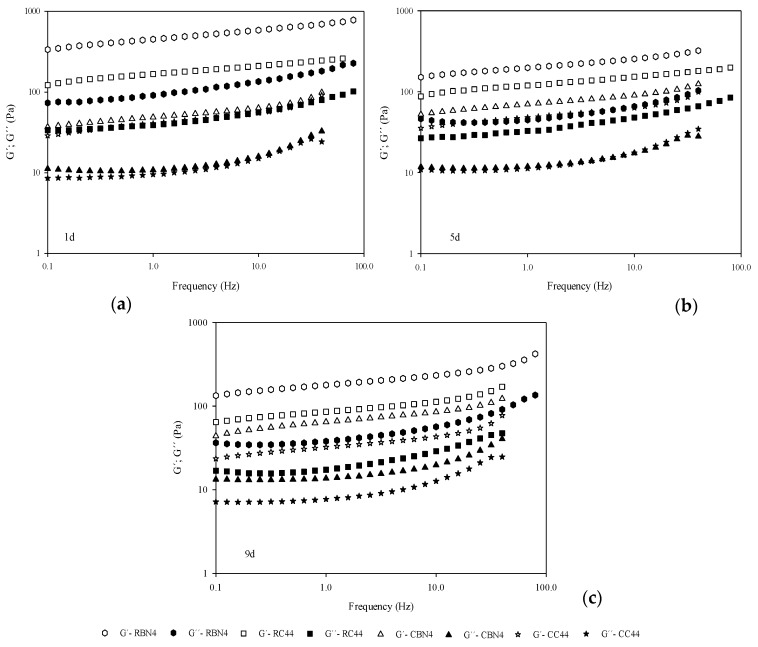
Mechanical spectra for four custard desserts (RBN4, RC44, CBN4, CC44) at (**a**) day 1, (**b**) day 5 and (**c**) day 9. Empty symbols for the storage modulus (G’) and filled symbols for the loss modulus (G’’).

Frequency sweeps at 20 °C for the selected four samples are shown in Figure 2, with both moduli, G’ and G’’ in the analyzed range at three different days of storage. In the four custards, the storage modulus was greater than the loss modulus, behavior that is characteristic of viscoelastic materials such as dispersions and gels [25,26,27]. The elastic response dominates the viscous one, for which it may be related to the structuring of molecules of the particular custard system, leading to this gel response. A weak dependence on frequency of both moduli is observed, as well as a function of the starch-hydrocolloid mixture in the gel structure. Similar mechanical spectra have been obtained by other researchers for hydrocolloid gels [7,9,28,29,30]. It could be expected that samples with lower content of flour would exhibit weaker gels. It may be observed that the addition of cooked flour (CBN4 and CC4) in the custard formulations, caused a decrease in G’ and G’’ (<100 Pa) at day 1, in comparison with the raw flour (RBN4 and RC4) with G’ > 100 Pa, as may be observed in Figure 2. This difference could be attributed to the presence of denatured protein and gelatinized starch due to the treatment in samples with cooked flour. 

Tan *δ* values, representing the ratio between G’’ and G’, were lower in formulations containing raw flour than samples containing cooked flour. With these results, the mechanical spectra confirmed the importance of starch and protein presence in generating a good structured dessert, as it has been confirmed by other authors. The gel strength of *κ*-carrageenan and milk protein systems, increased with both carrageenan and casein concentration [28,31]. In general, the effect of time on the desserts implied a loss of structuring, thus a decrease in both moduli was recorded at day 5 and 9, in which raw flour contributed to a weaker gel nature.

Several authors have reported values of the viscoelastic parameters at frequency of 0.5–1 Hz, which represents a value in which a human mouth begins to make structural changes. At that frequency of 0.5 Hz, the viscoelastic parameters for the four selected samples are included in Table 5, and, observing the magnitudes for both moduli, the most consistent or firmer product was the RBN4, with the highest moduli G’, G’’ and complex modulus G*. Alamprese and Mariotti [10] characterized the viscoelastic behavior of different puddings after storage at 4 °C for one day, reporting values of 105–442 Pa for G’, 1.73–68.5 Pa for G’’, and 12.6–445 Pa for G*. Torres et al. [30] showed values at 1 Hz of the same magnitude, for dairy dessert samples with and without inulin through storage time, and they reported an increase in these dynamic moduli. Zapata-Noreña et al. [4] also reported a range of 0 to 550 Pa for G’ and 0 to 100 Pa for G’’ for skimmed and whole milk custard desserts at various days (1, 3 and 6) also measured at 1 Hz, which are comparable to those recorded for our studied systems.

### 3.5. Textural Analysis

**Figure 3:**
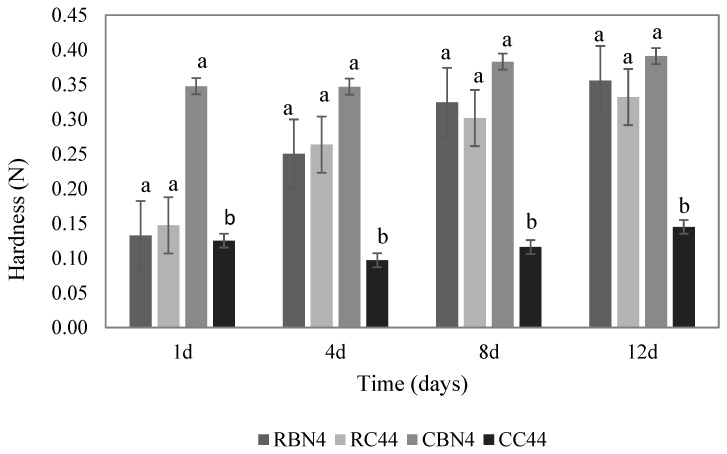
Average hardness for four custard desserts with raw and cooked chickpea flour (different letters indicating significant difference, *p* < 0.05 by Tukey test).

Texture profile analysis (TPA) of the same four selected systems, showed clear differences among dessert hardness (Figure 3) as the most important parameter from this test. The highest hardness corresponded to desserts prepared using both types of BN (raw and cooked), with hardness values of 0.133–0.391 N. The formulation with flour CC44 showed the lowest hardness, response that was consistent through storage. Thus, the type of flour had significant difference (*p* < 0.05) on this hardness parameter. In contrast, storage time had no significant effect (*p* > 0.05) on hardness. The other TPA parameters did not show important differences between samples.

The hardness values obtained in this study are similar to those reported by Szwajgier and Gustaw [21] for dairy desserts added with malt, whole and skim milk, who reported a range from 0.18 to 0.33 N. Alamri et al. [32] developed stronger puddings containing 0.14% of axseed and xanthan gums and reported hardness of 0.28 to 0.63 N, being higher than our studied custard systems.

### 3.6. Resistant Starch (RS) Quantification

Resistant starch is a natural component present in many foods. Some studies suggest that resistant starch have positive implications for human health, its fractions pass into the colon, which are fermented by the microorganisms producing mainly short chain fatty acids. Additionally, RS has a physiological effect similar to the dietary fiber, with functional properties and it has been observed that certain types of processing, increases the level of resistant starch [33,34]. 

**Figure 4:**
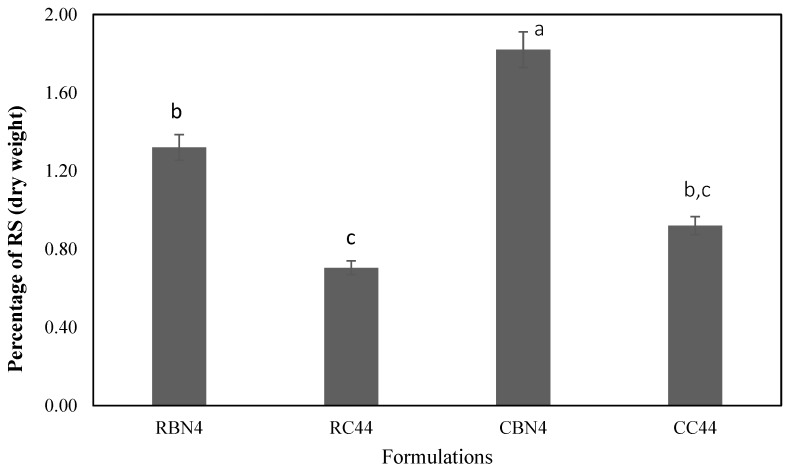
Percentage of resistant starch in four formulations of a dairy dessert (different letters indicating significant difference, *p* < 0.05 by Tukey test).

The amount of RS determined in the same selected four dairy desserts (added with the highest content of flour) presented a range of 0.75–1.84% (*w*/*w*, dry matter; Figure 4), and significant differences were observed. CBN4 had the highest value, followed by RBN4, CC44 and RC44. It is noted that the formulations with cooked flour showed a higher content of RS than raw flour samples. These values are in the range (0.8–4.2% *w*/*w*, dry matter) reported by Brumovsky et al. [35] for cassava, corn, potato and wheat starch. In the resistant starch values reported by Ratnayake et al. [36] for four peas, Osorio-Diaz et al. [37] for two bean varieties, and Tharanathan and Mahadevamma [38] for legumes, they mention that the heat treatment of seeds increased RS values due to retrogradation of amylose.

The presence of resistant starch has been detected in various foods such as bread, breakfast cereals, biscuits, corn mashed, potatoes and legumes. Pereira and Leonel [39] reported resistant starch in a cassava flour, ranging from 0.19 to 2.21% (dry weight).

## 4. Conclusions

A dairy dessert added with raw and cooked chickpea flour was formulated, prepared and characterized. Our results demonstrated that it is possible to produce and have a good alternative for generating dairy products with good properties and potential higher nutritional value. The incorporation of chickpea flour was successful, in which the custard properties were affected by the flour chickpea variety type, the level of concentration and the storage time in a different degree. From a physical-chemical point of view, all studied formulations showed good characteristics. In the rheological characterization, it was observed that both types of flour increased the viscosity of the products, which, in turn, contributed to a viscoelastic behavior. The determination of resistant starch in this type of products indicates an added value for the application of chickpea flour into other food products, particularly in the dairy dessert of this study.

## Figures and Tables

**Table 1:** Identification codes for custard systems.

Raw flour (R)	RBN1	RBN2	RBN3	RBN4
	RC41	RC42	RC43	RC44
Cooked flour (C)	CBN1	CBN2	CBN3	CBN4
	CC41	CC42	CC43	CC44
Chickpea flour (g)	8.3	9.3	10.3	11.3

BN = Blanco Noroeste; C4 = Costa 2004; Chickpea flour without treatment.

**Table 2:** Physicochemical properties of dairy dessert systems, 0 and 12 days.

Samples	Brix		pH		Acidity (g/L)		Syneresis (%)	
	Day 0	Day 12	Day 0	Day 12	Day 0	Day 12	Day 0	Day 12
RBN1	24.93 ± 0.04 ^abA^	21.27 ± 0.08 ^abB^	6.80 ± 0.01 ^aA^	6.98 ± 0.10 ^aA^	1.80 ± 0.17 ^cZ^	1.43 ± 0.01 ^cZ^	42.58 ^A^	46.75 ^B^
RBN2	24.07 ± 0.65 ^abA^	22.63 ± 0.13 ^abB^	6.70 ± 0.00 ^aA^	6.96 ± 0.05 ^aA^	1.77 ± 0.01 ^cZ^	1.13 ± 0.15 ^cZ^	46.43 ^A^	39.55 ^B^
RBN3	23.93 ± 0.06 ^abA^	21.83 ± 0.28 ^abB^	6.68 ± 0.01 ^aA^	7.12 ± 0.01 ^aA^	1.44 ± 0.06 ^cZ^	1.20 ± 0.03 ^cZ^	47.55 ^A^	27.30 ^B^
RBN4	24.13 ± 0.94 ^abA^	22.07 ± 0.17 ^abB^	6.74 ± 0.03 ^aA^	7.06 ± 0.23 ^aA^	1.56 ± 0.14 ^cZ^	1.10 ± 0.01 ^cZ^	34.19 ^A^	31.90 ^B^
RC41	25.10 ± 0.08 ^aA^	20.60 ± 0.27 ^aB^	6.86 ± 0.01 ^aA^	6.99 ± 0.32 ^aA^	1.80 ± 0.13 ^cZ^	1.40 ± 0.12 ^cZ^	46.52 ^A^	70.39 ^aB^
RC42	24.93 ± 0.17 ^aA^	21.10 ± 0.53 ^aB^	6.91 ± 0.03 ^aA^	6.96 ± 0.54 ^aA^	1.77 ± 0.16 ^cZ^	1.60 ± 0.07 ^cZ^	43.03 ^A^	70.24 ^aB^
RC43	26.73 ± 0.17 ^aA^	21.17 ± 0.11 ^aB^	6.95 ± 0.03 ^aA^	6.95 ± 0.21 ^aA^	1.44 ± 0.10 ^cZ^	1.20 ± 0.56 ^cZ^	39.35 ^A^	69.10 ^aB^
RC44	27.87 ± 0.06 ^aA^	21.20 ± 0.21 ^aB^	7.11 ±0.01 ^abA^	6.95 ± 0.12 ^aA^	1.56 ± 0.01 ^cZ^	1.37 ± 0.32 ^cZ^	28.35 ^A^	67.23 ^aB^
CBN1	20.40 ± 0.63 ^bcA^	20.10 ± 0.63 ^bcB^	6.97 ±0.03 ^abA^	6.67 ± 0.15 ^abA^	2.75 ± 0.16 ^aZ^	3.56 ± 0.31 ^aZ^	55.40 ^A^	50.75 ^B^
CBN2	21.53 ± 0.21 ^bcA^	21.00 ± 0.21 ^bcB^	7.01 ±0.01 ^abA^	6.61 ± 0.11 ^abA^	2.80 ± 0.07 ^aZ^	3.11 ± 0.34 ^aZ^	50.95 ^A^	46.55 ^B^
CBN3	21.91 ± 0.08 ^bcA^	20.50 ± 0.08 ^bcB^	6.97 ± 0.05 ^abA^	6.64 ± 0.01 ^abA^	2.82 ± 0.47 ^aZ^	3.15 ± 0.56 ^aZ^	44.85 ^A^	38.30 ^B^
CBN4	21.13 ± 0.10 ^bcA^	20.13 ± 0.10 ^bcB^	7.05 ± 0.21 ^abA^	6.67 ± 0.07 ^abA^	2.91 ± 0.21 ^aZ^	3.24 ± 0.78 ^aZ^	43.00 ^A^	50.90 ^B^
CC41	20.50 ± 0.27 ^cA^	20.00 ± 0.27 ^cB^	6.84 ± 0.17 ^bA^	6.43 ± 0.11 ^bA^	2.12 ± 0.06 ^bZ^	2.43 ± 0.04 ^bZ^	51.82 ^A^	42.30 ^B^
CC42	21.07 ± 0.02 ^cA^	20.10 ± 0.02 ^cB^	6.74 ± 0.51 ^bA^	6.35 ± 0.32 ^bA^	2.52 ± 0.12 ^bZ^	2.57 ± 0.02 ^bZ^	48.33 ^A^	38.52 ^B^
CC43	22.03 ± 0.05 ^cA^	21.23 ± 0.05 ^cB^	6.83 ± 0.01 ^bA^	6.44 ± 0.29 ^bA^	2.43 ± 0.32 ^bZ^	2.43 ± 0.09 ^bZ^	52.15 ^A^	40.39 ^B^
CC44	21.32 ± 0.01 ^cA^	21.32 ± 0.01 ^cB^	6.87 ± 0.40 ^bA^	6.45 ± 0.08 ^bA^	2.57 ± 0.09 ^bZ^	2.57 ± 0.11 ^bZ^	48.94 ^A^	39.43 ^B^

Values represent the mean of triplicate analysis ± standard deviation; Samples that do not share the same letter are significantly different (*p* < 0.05) by Tukey test. Lowercase letters represent differences in the type of flour, whereas capital letters correspond to differences in the storage time.

**Table 3:** Color parameters of dairy dessert systems, 0 and 12 days.

Samples	L*		a*		b*		∆E
	Day 0	Day 12	Day 0	Day 12	Day 0	Day 12	
RBN1	81.54 ± 0.73 ^a^	81.89 ± 0.52 ^a^	−3.98 ± 0.05 ^c^	−4.26 ± 0.07 ^c^	17.09 ± 0.47 ^C^	16.65 ± 0.44 ^C^	0.63 ^a^
RBN2	80.80 ± 0.14 ^a^	80.80 ± 0.15 ^a^	−3.88 ± 0.02 ^c^	−4.13 ± 0.22 ^c^	19.30 ± 0.41 ^C^	17.97 ± 0.20 ^C^	0.80 ^a^
RBN3	81.54 ± 0.83 ^a^	79.71 ± 1.28 ^a^	−4.16 ± 0.05 ^c^	−3.57 ± 0.14 ^c^	19.52 ± 0.08 ^C^	20.08 ± 0.02 ^C^	2.00 ^a^
RBN4	75.94 ± 1.21 ^a^	79.29 ± 0.29 ^a^	−3.43 ± 0.12 ^c^	−4.00 ± 0.16 ^c^	21.52 ± 0.61 ^C^	20.31 ± 1.98 ^C^	3.61 ^a^
RC41	83.29 ± 0.54 ^a^	80.63 ± 0.11 ^a^	−4.05 ± 0.45 ^c^	−4.20 ± 0.16 ^c^	18.17 ± 0.27 ^BC^	18.20 ± 1.19 ^BC^	3.53 ^ab^
RC42	82.80 ± 0.03 ^a^	79.77 ± 0.35 ^a^	−4.15 ± 0.10 ^c^	−4.07 ± 0.16 ^c^	19.74 ± 0.22 ^BC^	18.74 ± 0.38 ^BC^	4.02 ^ab^
RC43	82.93 ± 0.11 ^a^	78.69 ± 0.15 ^a^	−3.87 ± 0.10 ^c^	−3.93 ± 0.08^c^	20.67 ± 0.24 ^BC^	19.95 ± 0.55 ^BC^	4.89 ^ab^
RC44	81.37 ± 0.54 ^a^	78.42 ± 0.11 ^a^	−3.77 ± 0.05 ^c^	−4.04 ± 0.02 ^c^	21.35 ± 0.65 ^BC^	21.49 ± 0.43 ^BC^	4.53 ^ab^
CBN1	66.48 ± 0.31 ^b^	62.80 ± 0.76 ^b^	0.05 ± 0.05 ^a^	0.64 ± 0.01 ^a^	18.85 ± 0.25 ^AB^	21.10 ± 0.21 ^AB^	3.68 ^a^
CBN2	66.76 ± 0.65 ^b^	64.59 ± 0.32 ^b^	0.39 ± 0.06 ^a^	1.15 ± 0.14 ^a^	19.46 ± 0.38 ^AB^	22.00 ± 0.01 ^AB^	2.39 ^a^
CBN3	64.63 ± 0.96 ^b^	62.75 ± 1.01 ^b^	1.21 ± 0. 27 ^a^	1.65 ± 0.02 ^a^	20.11 ± 0.21 ^AB^	22.50 ± 0.12 ^AB^	2.01 ^a^
CBN4	64.11 ± 0.59 ^b^	63.56 ± 0.78 ^b^	1.35 ± 0.03 ^a^	2.89 ± 0.09 ^a^	20.22 ± 0.10 ^AB^	23.30 ± 0.27 ^AB^	0.63 ^a^
CC41	73.46 ± 0.83 ^c^	69.26 ± 0.22 ^c^	−0.89 ± 0.38 ^b^	−1.19 ± 0.04 ^b^	19.28 ± 0.12 ^A^	17.30 ± 0.07 ^A^	4.65 ^b^
CC42	76.01 ± 0.78 ^c^	69.95 ± 0.64 ^c^	−0.55 ± 0.21 ^b^	−0.75 ± 0.07 ^b^	20.05 ± 0.32 ^A^	18.19 ± 0.34 ^A^	6.34 ^b^
CC43	75.95 ± 0.76 ^c^	67.47 ± 0.37 ^c^	−0.41 ± 0.01 ^b^	−0.65 ± 0.00 ^b^	20.38 ± 0.19 ^A^	17.97 ± 0.03 ^A^	8.86 ^b^
CC44	76.23 ± 0.31 ^c^	65.99 ± 0.01 ^c^	−0.55 ± 0.07 ^b^	−0.34 ± 0.07 ^b^	21.36 ± 0.57 ^A^	17.46 ± 0.13 ^A^	10.95 ^b^

Values represent the mean of triplicate analysis ± standard deviation. Samples that do not share the same letter are significantly different (*p* < 0.05), Tukey test. Lowercase letters represent differences in the type of flour and capital letters correspond to concentration. L*, a*, b* are the color parameters.

**Table 4:** Rheological parameters for dairy dessert systems from the best fittings to three flow models.

Muestras	Day 0							Day 12						
	PL		HB			BP		PL		HB			BP	
	*n*	*K* (Pa·s^n^)	*τ*_0_ (Pa)	*n*	*K* (Pa·s^n^)	*τ*_0_ (Pa)	*ɳ*_p_ (Pa·s)	*n*	*K* (Pa·s^n^)	*τ*_0_ (Pa)	*n*	*K* (Pa·s^n^)	*τ*_0_ (Pa)	*ɳ*_p_ (Pa·s)
RBN1	0.31	4.13						0.36	2.12					
RBN2						4.97	0.15						8.14	0.42
RBN3						36.02	1.13						32.35	0.12
RBN4			76.50	0.59	5.52					55.72	0.51	0.09		
RC41			6.41	0.61	1.20					1.50	0.54	0.58		
RC42			18.81	0.62	3.45					14.45	0.50	1.54		
RC43			36.08	0.58	4.73					12.07	0.70	2.35		
RC44						56.18	1.39						72.29	2.28
CBN1	0.26	1.13						0.28	1.16					
CBN2	0.23	2.42						0.29	1.82					
CBN3	0.18	11.76						0.36	2.30					
CBN4	0.37	3.28						0.36	2.91					
CC41			2.78	0.28	0.68					2.24	0.74	0.45		
CC42						4.05	0.08						3.20	0.06
CC43			2.69	0.64	0.33					1.96	0.74	0.43		
CC44			2.81	0.61	0.54					2.26	0.62	0.41		

PL: Power law; HB: Herschel and Bulkley; BP: Bingham Plastic. *K* = consistency coefficient (Pa·s^n^); *n* = flow behavior index (dimensionless); *τ*_0_ = yield stress (Pa); and *ɳ*_p_ = plastic viscosity (Pa·s).

**Table 5:** Values (average and standard deviation) of the viscoelastic parameters, calculated from custard mechanical spectra at a frequency of 0.5 Hz at day 1.

Formulation	G’ (Pa)	G’’ (Pa)	Tan *δ*	G* (Pa)
RBN4	418 ± 31 ^a^	83.4 ± 3.3 ^a^	0.200 ^a^	426 ± 16 ^a^
RC44	156 ± 23 ^ab^	36.9 ± 4.6 ^ab^	0.235 ^a^	160 ± 12 ^ab^
CBN4	45 ± 2.2 ^b^	10.5 ± 3.8 ^b^	0.231 ^a^	46 ± 2.32 ^b^
CC44	37 ± 5.9 ^b^	9.0 ± 2.2 ^b^	0.239 ^a^	38 ± 6.05 ^b^

Data followed by different letters in the same column are significantly different (*p* < 0.05) by Tukey test. G’: storage modulus; G’’: loss modulus; Tan *δ : G’’/G’*; G*: complex modulus.

## Abbreviations

RC4	raw flour Costa 2004
RBN	raw flour Blanco Noroeste
CC4	cooked flour Costa 2004
CBN	cooked flour Blanco Noroeste
PL	power law model
HB	Herschel–Bulkley model
PB	Bingham plastic model
